# A BELGIAN VIEW ON BEING RETIRED BUT NOT OUT OF WORK

**DOI:** 10.1093/geroni/igz038.079

**Published:** 2019-11-08

**Authors:** Sarah Dury

**Affiliations:** Vrije Universiteit Brussel, Brussels, Belgium

## Abstract

Retirement is no longer merely the end of a productive life. This changing nature of retirement challenges the common definitions of retirement – that define retirement primarily by what it is not (i.e. no longer working). The aim of this paper is to gain insight into the activity patterns of individuals who recently retired from a full-time job in relation with their well-being. We use data from a qualitative study in which we conducted semi-structured interviews with 45 individuals who retired one to two years ago in Belgium. We used a hybrid approach of inductive and deductive thematic analysis. Our findings demonstrate that most of the people who are retired from their full-time job remain active within society. First, productive activities, including work and civic engagement. Second, consumer-oriented activities comprising leisure and social contacts. The results suggest that being active, regardless of the type of activity, contributes to well-being.

